# Effect of storytelling on learning about the institutionalization of Unified Health System: randomized controlled trial

**DOI:** 10.1590/0034-7167-2025-0094

**Published:** 2026-02-23

**Authors:** Államy Danilo Moura e Silva, Andreia Rodrigues Moura da Costa Valle, José Wicto Pereira Borges, Thereza Maria Magalhães Moreira, Maria Augusta Rocha Bezerra, Danielle Christine Moura dos Santos

**Affiliations:** I Universidade Estadual do Piauí. Teresina, Piauí, Brazil.; II Universidade Federal do Piauí. Teresina, Piauí, Brazil.; III Universidade Estadual do Ceará. Fortaleza, Ceará, Brazil.; IV Universidade do Pernambuco. Recife, Pernambuco, Brazil.

**Keywords:** Education, Narrative Medicine, Learning, Health Systems, Institutionalization

## Abstract

**Objectives:**

to evaluate the effect of using storytelling as a teaching strategy on nursing students’ learning about the Brazilian Unified Health System.

**Methods:**

a randomized controlled trial with 48 nursing students, divided into intervention (storytelling class) and control (traditional class) groups, conducted remotely. Learning was assessed through pre-test, immediate post-test, and post-test 15 days later. Statistical analyses included t-tests, Chi-square, McNemar’s, and Cochran’s Q tests.

**Results:**

both groups showed significant learning improvement in the immediate post-test (p<0.001). In the 15-day post-test, the Intervention Group demonstrated higher retention of knowledge, with scores ≥70% significantly higher than the Control Group (p<0.001).

**Conclusions:**

storytelling had a positive and lasting impact on learning about the Unified Health System, promoting an engaging and participatory teaching environment. This strategy aligns with the shift in nursing education towards more dynamic methodologies.

## INTRODUCTION

Within the current dimensions of nursing education, there is an ongoing process of transforming traditional knowledge and practices into innovative approaches^(1–3)^. Recognizing the attitudes and actions of those involved in this construction, along with the sociocultural atmosphere, is crucial for developing nursing students’ professional identity^(4,5)^. However, despite governmental and societal efforts to align nursing education with the principles of the Unified Health System (SUS) in Brazil, many Higher Education Institutions (HEIs) still rely on traditional curricular structures. These structures often use teaching methodologies and educational materials that provide little stimulus for critical thinking, reflection, creativity, and proposing changes that address the needs and cultural values of students^(6,7)^.

This gap between health education, societal needs, and the reality of public health and SUS creates challenges in the teaching and learning process. The quality of healthcare is closely related to the education process, and a pedagogical approach emphasizing participatory and reflective teaching and learning can be a determinant for improving healthcare, especially within the scope of SUS^(8,9)^.

The historical concern for nursing education and human resources in nursing predates current publications, with early Brazilian nursing leaders expressing unease about issues related to technical-scientific and ethical-political training. Nursing, as a profession, has the opportunity to creatively and autonomously contribute to different levels of healthcare, aligning with the foundational principles of SUS^(10,11)^. Students play an active role in shaping their education and influencing society. The aim is to train healthcare professionals to collaborate with interdisciplinary healthcare providers according to the needs of SUS users.

In this context, Paulo Freire’s reflections on education as a dynamic and continuous process of knowledge construction become relevant, emphasizing the role of free thought and critical-reflexive consciousness in fostering personal and professional engagement^(12)^. As part of the educational paradigm shift, Active Methodologies (AM) promote critical-reflexive learning by stimulating student involvement in knowledge-seeking^(13)^. Storytelling, as an academic tool, presents fictional or real problematizing cases to engage learners, relying on four key elements: attention, motivation, emotions, and student experiences, which are essential for effective learning^(14,15)^.

This study acknowledges the challenges of teaching a new generation of students increasingly engaged with digital technology, which creates expectations for immediate and dynamic learning. Traditional classroom models often fail to meet these demands, making theoretical topics like the history, institutionalization, and regulation of the SUS tiresome. Within the framework of active learning methodologies, the study assumes that innovative tools can enhance teaching practices and support student-centered learning. Therefore, it explores and tests the impact of storytelling as a strategy to teach and engage students with the historical and institutional aspects of the SUS and its relevance to Brazilian public health.

## OBJECTIVES

To evaluate the effect of using storytelling as a teaching strategy on nursing students’ learning about the history and institutionalization of the Brazilian Unified Health System.

## METHODS

### Ethical aspects

The study was conducted in accordance with national and international ethical guidelines and approved by the Research Ethics Committee of the Universidade Federal do Piauí (UFPI), whose opinion is attached to this submission. The Free and Informed Consent Form was obtained from all individuals involved in the study online.

### Study design, period and location

A randomized controlled trial (RCT) was conducted to evaluate the effect of storytelling on nursing students’ learning about the history and institutionalization of the Unified Health System (SUS). The intervention group received a storytelling-based lecture, while the control group attended a traditional lecture on the same topic. The study took place in February 2022 at the Nursing Course at the Federal University of Piauí (UFPI), in Teresina, Piauí, adhering to the guidelines of the Consolidated Standards of Reporting Trials (CONSORT).

### Population or sample, inclusion and exclusion criteria

Participants were undergraduate nursing students from the Federal University of Piauí. The study included all students enrolled in the Primary Health Care course in the second semester of the Bachelor of Nursing program. Inclusion criteria were regular enrollment in the second semester and students aged 18 years or older. Exclusion criteria included any plans to be absent in the next 15 days. After applying the inclusion and exclusion criteria, a total of 51 students comprised the sample, with 48 completing the study.

### Study protocol

Simple randomization was used to assign students to the Intervention Group (IG) and Control Group (CG), conducted by an external member using the Research Randomizer software in a 1:1 ratio. Due to COVID-19 restrictions, the study was conducted remotely. To achieve its objectives, a fictional story was developed using the storytelling methodology^(16)^, incorporating content on the history and institutionalization of SUS. This narrative served as the foundation for the Assessment Tool (TVA), ensuring alignment between the learning evaluation and the story’s topics (Table 1).

**Table 1:** Contents related to the history and institutionalization of Unified Health System, Teresina, Piauí, Brazil, 2025

Contents related to the history and institutionalization of Unified Health System
• Sanitarism in the Old Republic; • Oswaldo Cruz and the campaign model; • Vaccine Revolt; • Carlos Chagas and changes in the campaign model; • Eloy Chaves Law of 1923; • Retirement and Pension Funds (CAP); • Evolution of public health in Brazil; • Vargas era; • Retirement and Pension Institutes (IAP); • National Social Security Institute (INPS); • International Conferences on Health Promotion; • Ottawa Charter; • VIII National Health Conference; • Federal Constitution of 1988; • Creation of the Unified Health System; • Law No. 8,080/1990; • Law No. 8,142/1990; • SUS regulations and principles; • Social control of the Unified Health System: Conferences and Health Councils; • Basic Operational Standards; • Health Care Operational Standards; • Decree 7,508/2011.

For this study, the narrative revolves around a protagonist who interacts with other characters throughout the story. The central character is portrayed as a second-semester nursing student named Elina, who, due to her inquisitive nature and growing interest in public health and the Brazilian Unified Health System, begins drawing various analogies between historical events and her own life experiences. Consequently, she embarks on a journey of exploration and knowledge. The fictional narrative is titled “Elina’s Discoveries”.

The instrument consisted of 15 objective questions, each offering five answer choices, with one being correct.

The content of the story was validated by experts in the public health field. Expert selection followed criteria adapted from Jasper^(17)^, requiring professionals to meet at least two of Jasper’s suggested requirements to be considered specialists in the area. Experts were recruited through convenience sampling, using the snowball method, where eligible judges were asked to recommend other potential specialists. The exclusion criterion was incomplete completion of the content assessment instruments.

The experts’ resumes were reviewed to identify potential specialists, and an electronic form was sent to them, including an invitation letter and an Informed Consent Form for specialists. The Instrument for the Validation of Educational Content in Health (IVCES) was also sent, which was constructed and validated by Leite et al^(18)^. This instrument assesses educational content in health across three domains (objectives, structure/presentation, and relevance) using a Likert scale.

Before evaluating the storytelling and completing the IVCES, experts were briefed on this teaching strategy through a message included in Google Forms and a judge characterization form. Additionally, the Content Validation Instrument for the Learning Verification Test was sent to the experts. An assessment was conducted for each question, covering objectivity, comprehension, and relevance, based on Bellan’s^(19)^ multiple-choice question validation instrument.

The experts’ feedback was incorporated into the final version of the storytelling, adhering to the same criteria used in the initial construction. The judges did not provide suggestions for the TVA instrument, so the original version remained unchanged.

To assess expert agreement on the data collection instrument’s domains, the Content Validity Index (CVI) was calculated in three forms: I-CVI, measuring agreement on individual items; S-CVI/AVE, representing the proportion of agreed-upon items per judge; and S-CVI, calculated as the average of S-CVI/AVE.

Data were stored in Microsoft Office Excel 2016, with double-entry applied for error correction and database cleaning. Statistical analysis was conducted using R software, version 4.0.3, in a Windows environment.

The descriptive analysis of the expert’ profile involved calculating absolute and relative frequencies. For validation stage data analysis, the Content Validity Index was used, considering an item valid if the expert judges’ agreement proportion was equal to or greater than 0.80 (80%). The binomial test was used to assess whether the judges’ agreement proportion for a particular item was statistically equal to the established value, with a significance level of 5% and a 95% confidence interval for all tests.

In this phase, the analysis focused on the impact of using the storytelling methodology to enhance learning about the history and institutionalization of the Brazilian Unified Health System. A RCT was conducted, employing the storytelling educational strategy for the intervention group and exposing the control group to traditional didactic lectures^(20)^.

**Figure 1:**
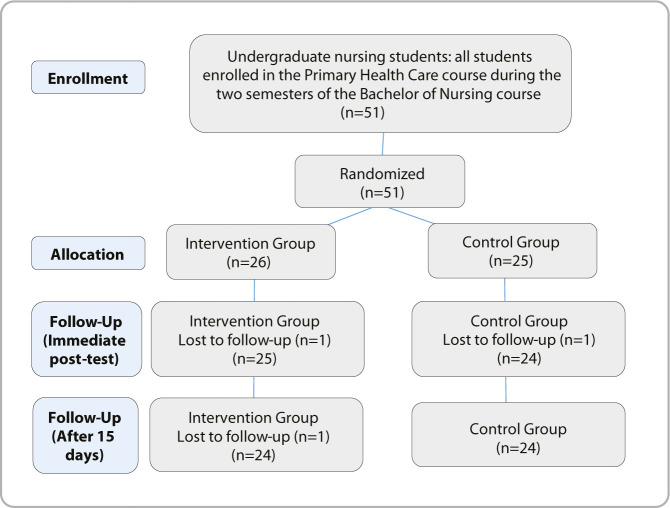
Consolidated Standards of Reporting Trials, Teresina, Piauí, Brazil, 2025

The study involved a total of 51 students participating, with 48 completing the study (24 in IG and 24 in CG) (Figure 1).

Randomization is inherent to experimental studies, ensuring the even distribution of participant characteristics between control and experimental groups. Simple randomization was adopted for this study. After departmental approval, authorization was obtained from the professor of the Primary Health Care discipline. A designated member, external to the study team, conducted the randomization using the Research Randomizer program (Version 4.0). The randomization process involved using an enumerated attendance list, assigning numbers based on the order of attendance. The sequence was determined by the Research Randomizer program, with Set 1 representing the control group and Set 2 the intervention group.

### Analysis of results and statistics

As for the outcome variable, students’ learning about the history and institutionalization of the Brazilian Unified Health System was measured using a data collection instrument. Learning, in this context, is defined based on empiricism and experiences, where senses cognitively associate to form knowledge. Knowledge, despite having a personal and subjective component, includes explicit elements that can be transmitted through language, involving cognitive structures that allow information assimilation and action generation^(21,22)^.

The assessment of learning was based on the number of correct answers in the data collection instrument specifically constructed and validated for this purpose. Learning was considered satisfactory when participants achieved a score equal to or above 70% on the data collection instrument. This criterion aligns with Brazilian universities’ grading standards, where 70% is often the passing threshold. Hence, in this study, 70% of the 15 questions were set as the reference for defining satisfactory outcomes.

The principal researcher solely presented the storytelling-based class on the history and institutionalization of SUS to the IG. The traditional didactic lecture, presented to the CG, was conducted by the course professor to minimize bias due to potential conflicts of interest.

Due to the COVID-19 pandemic, the study was conducted via Google Meet to ensure participant and researcher safety. The process included: I - Pre-test, where participants completed a SUS knowledge assessment via Google Forms (45-minute limit); II - Class Development, with the CG attending a 2-hour lecture and the IG receiving a storytelling-based lesson on the same content; and III - Post-test, administered immediately and repeated after 15 days to assess knowledge retention.

Blinding was challenging due to ethical considerations; while the principal researcher knew participant allocation, pre- and post-test evaluators remained blinded. The Intervention Group attended a storytelling-based class via Google Meet, with narration, screen projection, and discussions using Mentimeter for interactive engagement. The Control Group received a traditional lecture on SUS history and institutionalization, with interaction through microphone-based. Fifteen days later, both groups participated in a remote post-test via Google Meet to assess knowledge retention, with coded participants to ensure blinding during evaluations.

## RESULTS

The story outlines the key aspects of public health in Brazil from the early 20th century, while the protagonist is a second-year nursing student named Elina, who, due to her curiosity and growing interest in public health and SUS-related themes, begins drawing parallels between historical and current events on the topic with her personal life experiences. Thus, she immerses herself in a world of discovery and knowledge. A nursing student was intentionally chosen as the main character to nurture a connection with the narrative context and allow students to reflect on their personal experiences of learning about public healthcare.

Furthermore, the story was adapted to reflect the context of the current COVID-19 pandemic. For example, the characters in the story adhered to social distancing and endured restrictive measures which aligned with the reality of the students’ experiences. Other characters in the story include Cristian, an undergraduate nursing student and Elina’s classmate; Professor Ana, a nurse and professor in the nursing undergraduate program that Elina and Cristian attended; Dona Maria Rendeira, a resident of Elina’s community/neighborhood; and Mr. Paulo from the local market, a resident of Cristian’s neighborhood.

Out of the 14 experts, 12 agreed on all items in the objective domains, structure/presentation, and relevance. The S-CVI for content validation was 0.99. Regarding the I-CVI, of the 18 assessed items, 16 received approval from all experts (I-CVI = 1.0). Two items within the structure/presentation domain did not receive approval from all experts. One expert disagreed with the item related to appropriate language for educational material (I-CVI = 0.92), and two judges disagreed with the item concerning clarifying information (I-CVI = 0.85).

Regarding the suggested modifications made by the judges, these were associated with the item referring to appropriate language for educational material. It was recommended to exclude terms that could make the text “childish” and, thus, make it more suitable for the target audience. This suggestion was accepted, and expressions that would give a childlike focus were changed.

As for the use of storytelling, translated as “*contação de histórias*” in Portuguese, it is a pedagogical approach present in early childhood education practices, serving as a facilitator for addressing various themes in this stage of schooling. However, storytelling is also an ancient practice in civilizations, providing humanity with the opportunity to systematize and express ideas and understand the world^(23)^. Therefore, storytelling is used as a strategy for educational purposes in general.

Another suggested modification was related to the “clarifying information” item, where it was recommended to include the dates of the creation of laws 8,080/90 and 8,142 in the storytelling text. However, this change was not accepted, as some information was omitted from the text to encourage discussion during the presentation of the content by the facilitator.

A total of 51 students were included in the study (25 in the control group and 26 in the intervention group), of which 3 were discontinued (1 from the control group and 2 from the intervention group) as they did not participate in the immediate post-test after the lessons and did not respond to the post-test within 15 days. Thus, the final study sample consisted of 48 participants (24 in the control group and 24 in the intervention group) (Table 2).

**Table 2:** Characterization and homogeneity of students participating in the intervention and control groups, regarding demographic, economic data and prior knowledge of the Unified Health System, Teresina, Piauí, Brazil, 2025

Categorical variables	Intervention group n (%)	Control group n (%)	p
**Sex**			
Masculine	9 (34.6)	8 (32.0)	0.843^a^
Feminine	17 (65.4)	17 (68.0)	
**Marital status**			
Single	24 (92.3)	25 (96.0)	0.515^b^
Married	2 (7.7)	1 (4.0)	
**Sources of income**			
Allowance	15 (57.7)	15 (60.0)	0.000^b^
I have no income	9 (34.6)	7 (28.0)	
Own work	2 (7.7)	3 (12.0)	
**Have you fully or partially completed any other course in the healthcare field?**			
No	26 (100)	25 (100)	1.000^a^
**Have you ever taken a course that dealt with the history and institutionalization of the SUS?**			
No	26 (100)	25 (100)	1.000^a^

Numeric variables	Intervention group Average	Control group Average	*p*
**Age (years)**	20.77 (2.141)	20.40 (1.708)	0.500^c^
**Income of everyone in the house**	3926.92 (1575,642)	3808 (1841.403)	0.805^c^
	**Median**	**Median**	
**Age (years)**	21 (19.75-22)	20 (19-22)	0.680^d^
**Family composition**	3 (2-4)	3 (2-4)	0.867^d^
**Income of everyone in the house**	4500 (2300-5000)	4000 (2000-5000)	0.630^d^

a Chi-square test;

b Fisher's Exact Test;

c Student t test for independent samples;

d Mann-Whitney U test.

Regarding homogeneity, the intervention and control groups were considered homogeneous concerning the investigated variables. Additionally, no participant reported having partially or fully completed another health-related course, nor had they taken any course covering the history and institutionalization of the Unified Health System.

The intergroup comparisons regarding student learning are presented in chronological order of measurements (pre-test, immediate post-test, and post-test after 15 days).

Regarding the pre-test, there was similarity in correct answers between the groups in 14 questions. The question where a significant difference occurred, with a significantly higher number of correct answers in the intervention group, was question 4, which deals with Health Promotion and the Ottawa Charter (Table 3).

**Table 3:** Comparison of correct answers to questions between participants in the intervention and control groups in the pre-test, Teresina, Piauí, Brazil, 2025

Contents related to the history and institutionalization of Unified Health System	Pre-test		
	Intervention group n (%)	Control group n (%)	*p* ^a^
Q1 - Sanitarism in the Old Republic; Vaccine Revolt; campaign model	15 (57.5)	10 (40.0)	0.063
Q2 - Eloy Chaves Law of 1923; Retirement and Pension Funds (CAP); Retirement and Pension Institutes (IAP)	5 (19.2)	6 (24.0)	1.000
Q3 - Historical evolution of public health in Brazil; National Social Security Institute (INPS); VIII National Health Conference; Federal Constitution of 1988	6 (23.1)	6 (24.0)	1.000
Q4 - First International Conference on Health Promotion; Letter from Ottawa	17 (65.4)	11 (44.0)	0.031
Q5 - VIII National Health Conference; Federal Constitution of 1988; Creation of the SUS	9 (34.6)	5 (20.0)	0.125
Q6 - Article 198 of the Federal Constitution	1 (3.8)	3 (12.0)	0.500
Q7 - Law No. 8,080/1990; Unified Health System principles	4 (15.4)	4 (16.0)	1.000
Q8 - Article 6 of Law No. 8,080/1990	8 (30.8)	6 (24.0)	0.500
Q9 – Unified Health System Principles; Equity	4 (15.4)	4 (16.0)	1.000
Q10 – Unified Health System Principles; Universality	6 (23.1)	8 (32.0)	0.500
Q11 - Law nº 8,142/1990	5 (19.2)	7 (28.0)	0.500
Q12 - Health Conferences	8 (30.8)	6 (24.0)	0.500
Q13 - Health Advice	6 (23.1)	7 (28.0)	1.000
Q14 - Basic Operational Standard 93 and 96; Health Care Operational Standards 2001 and 2002	2 (7.7)	3 (12.0)	1.000
Q15 - Decree 7,508/2011	5 (19.2)	7 (28.0)	0.500

a *Chi-square test.*

In the immediate post-tests, there were also similarities between the groups in most of the evaluated questions (Table 4). The immediate post-test showed a difference between the groups (higher correct answers in the intervention group) in questions 1 and 3, related to sanitarism in the Old Republic/Vaccine Revolt and the historical evolution of public health and the INPS, respectively.

**Table 4:** Comparisons of correct answers to questions between participants in the intervention and control groups, in the immediate post-test, Teresina, Piauí, Brazil, 2025

Contents related to the history and institutionalization of Unified Health System	Control group n (%)	Intervention group n (%)	*p* ^a^
Q1 - Sanitarism in the Old Republic; Vaccine Revolt; campaign model	15 (62.5)	22 (88.0)	0.016
Q2 - Eloy Chaves Law of 1923; Retirement and Pension Funds (CAP); Retirement and Pension Institutes (IAP)	18 (75.0)	22 (88.0)	0.125
Q3 - Historical evolution of public health in Brazil; National Social Security Institute (INPS); VIII National Health Conference; Federal Constitution of 1988	16 (66.7)	23 (92.0)	0.016
Q4 - First International Conference on Health Promotion; Letter from Ottawa	17 (70.8)	21 (84.0)	0.125
Q5 - VIII National Health Conference; Federal Constitution of 1988; Creation of the Unified Health System	18 (75.0)	23 (92.0)	0.063
Q6 - Article 198 of the Federal Constitution	16 (66.7)	21 (84.0)	0.063
Q7 - Law No. 8.080/1990; Unified Health System principles	19 (79.2)	22 (88.0)	0.250
Q8 - Article 6 of Law No. 8.080/1990	16 (66.7)	21 (84.0)	0.063
Q9 – Unified Health System Principles; Equity	18(75.0)	22 (88.0)	0.125
Q10 – Unified Health System Principles; Universality	17 (70.8)	22 (88.0)	0.063
Q11 - Law nº 8.142/1990	21 (87.5)	23 (92.0)	0.500
Q12 - Health Conferences	17 (70.8)	20 (80.0)	0.250
Q13 - Health Advice	17 (70.8)	22 (88.0)	0.063
Q14 - Basic Operational Standard 93 and 96; Health Care Operational Standards 2001 and 2002	18 (75.0)	20 (80.0)	0.500
Q15 - Decree 7.508/2011	17 (70.8)	22 (88.0)	0.063

a *Chi-square test.*

In the post-test after 15 days, there was a greater difference in correct answers, with questions 1 and 3 mentioned earlier maintaining their distinctions. Additionally, significant differences were observed in the correct answers for question 5, concerning the Eighth National Health Conference, the 1988 Federal Constitution, and the creation of SUS. Furthermore, question 8, which addressed Article 6 of Law No. 8,080/1990, as well as questions 9 and 10, covering the principles of equity and universality in SUS, respectively, showed notable discrepancies (Table 5).

**Table 5:** Comparisons of correct answers to questions between participants in the intervention and control groups, in the post-test after 15 days, Teresina, Piauí, Brazil, 2025

	Correct answers to questions ≥70%		*p* ^a^
	Intervention group n (%)	Control group n (%)	
**Pre-test**	0 (0.0)	0 (0.0)	§
**Post-test**	15 (62.5)	11 (45.8)	0.125
**After 15 days**	15 (62.5)	2 (8.0)	<0.001

The pre-test showed no significant differences between the intervention and control groups, indicating similar prior knowledge. Both groups improved in the immediate post-test, demonstrating the effectiveness of storytelling and lecture methods, though the intervention group had more correct answers. Notably, after 15 days, the storytelling group showed a statistically significant learning advantage.

## DISCUSSION

The multiple-choice questions serve as an assessment method, aiming to evaluate student understanding of the course objectives. These questions include a problem or case description with five alternative answers, one correct and others equally plausible. However, few professors adequately develop this activity^(24)^. The study evaluates learning, guiding decisions on effective learning methods and providing insights into educational efficacy through the mapping of students’ knowledge acquisition process. Expert judges considered the instrument valid based on objectivity, comprehension, and relevance (experts’ agreement > 80%).

Given the academic profile and inclusion criteria (second-semester nursing students without prior exposure to SUS-related topics), participants had low prior knowledge. Both groups (GI and GC) had low pre-test scores in the learning assessment instrument. The nursing discipline in Primary Health Care, part of the second semester, served as the study’s setting. The curriculum aligns with the National Health Council’s guidelines for nursing education, emphasizing learning dimensions: to be, to do, to live together, and to know.

Training programs focusing on the expanded concept of health in SUS require teaching methodologies reflecting historical, political, economic, and social contexts related to the health-disease process. After the study’s proposed classes, both groups showed increased learning in the immediate post-test. The enduring significance of SUS in professional practice highlights its role as a significant social achievement grounded in principles such as universality, equity, and comprehensiveness, structured by organizational directives like decentralization, regionalization, hierarchy, and popular participation^(25)^.

The foundations of SUS are linked to the role of higher education institutions in training professionals aligned with the current care model, meeting public health system requirements in Brazil. The consolidation of SUS demands identification, formulation, and strengthening of actions in the human resources field^(26)^. Compared to other problem-solving and critical-thinking strategies, storytelling ensures more long-lasting learning. This aligns with andragogy, a science that emphasizes the adult learning process, taking into account psychological, biological and social aspects, which among its basic premises is the motivation to learn from cases and problem solving^(27)^.

Freire emphasizes the transformative role of educators using problematization, highlighting the need for constant dialogue between educators and students. The use of active methods requires a willingness to learn and a commitment to the meaningful presentation of new knowledge. Thus, storytelling as a teaching strategy is aligned with the scientific evidence that supports the use of active methodologies in the collective construction of knowledge, and rooted in Freirean thought, it favors meaningful, ethical and civic learning, essential for the training of critical professionals committed to transforming reality^(28)^.

Although storytelling is increasingly practiced by nursing educators, it often remains implicit. Further attention in nursing research is required to establish storytelling as a science, contributing to the development of the nursing identity and a connection to interprofessional practice^(29,30)^.

The study’s elements and outcomes offer new possibilities for teaching and learning about SUS history and institutionalization. It emphasizes the importance of teacher training as a social practice that can change lives.

### Study limitations

The main limitation of this study is that the evaluation of interventions using storytelling, combined with the generative words dynamic and expository-dialogic classes, was conducted in a remote environment, despite the recognition of virtual learning by Brazil’s Ministry of Education. Therefore, future studies should replicate this methodology and explore the effects of storytelling combined with strategies that enhance student protagonism in different settings, emphasizing nursing practice through in-person interventions.

### Contributions to the area

Through the elements developed in this study and the outcomes observed, it will be possible to disseminate new teaching and learning possibilities regarding the history and institutionalization of the SUS. This can be achieved by implementing teaching, research, and extension strategies that may contribute to paradigm shifts in the training process of nurses, overcoming the traditional way of teaching and learning. This highlights the importance of teacher training as a social practice that transforms lives.

## CONCLUSIONS

This study successfully achieved its goals by developing storytelling and a data collection instrument related to the history and institutionalization of the Brazilian public health system. Content validation was conducted with experts in public and community health. The study compared learning outcomes before and after the use of storytelling, before and after traditional expositional classes, and between students exposed to storytelling and those in traditional expositional classes.

In intragroup comparisons, both the storytelling group (GI) and the traditional expositional class group (GC) showed a significant increase in learning about the history and institutionalization of SUS in the immediate post-test (GI and GC: <0.001). Thus, both storytelling and traditional expositional classes were effective in improving students’ learning. In intergroup comparisons, the proportions of total TVA scores were similar in the pre-test and immediate post-test, while in the post-test 15 days later, the GI’s score proportion was superior to that of the GC.

The results showed that storytelling had a superior effect on learning about the history and institutionalization of SUS. While there was no significant difference between groups in the pre-test (§) and immediate post-test (p=0.125), the post-test after 15 days revealed a significantly higher proportion of IG participants scoring ≥70% (p<0.001). This confirms that storytelling as a teaching strategy has scientific support and a longer-lasting learning effect, validating its positive impact on nursing students’ learning.

## Data Availability

The research data are available within the article.
